# Results of a Community-Based Screening Program for Chlamydia trachomatis Genital Infection in Young People Aged 18–25 Years

**DOI:** 10.7759/cureus.46916

**Published:** 2023-10-12

**Authors:** Noemi Espies, Joan Fernandez, Elena Justribo, Jesus Aramburu, Albert Bernet, Alicia Marquez, Pere Godoy, Oriol Yuguero Torres

**Affiliations:** 1 Primary Care, Catalan Health Institute, Lleida, ESP; 2 ERLab, Research in Emergency Medicine, Biomedical Research Institute of Lleida (IRBLLEIDA), Lleida, ESP; 3 Medicine and Surgery, University of Lleida, Lleida, ESP; 4 Research in Clinical Microbiology and Antibiotic Resistance, Biomedical Research Institute of Lleida (IRBLLEIDA), Lleida, ESP; 5 Applied Epidemiology, Biomedical Research Institute of Lleida (IRBLLEIDA), Lleida, ESP

**Keywords:** community health, sexual transmitted infections, youth health, screening, chlamydia trachomatis

## Abstract

Introduction and aim

*Chlamydia trachomatis *(CT) cases have increased in the last decade. The aim of the study was to assess the prevalence of CT genital infection in asymptomatic, sexually active young people and determine whether a community screening program would be effective in reducing the number of cases.

Methods

A descriptive cross-sectional studyof consecutive inclusion of asymptomatic people aged 18-25 years between September 2021 and May 2022. Community interventions in high schools, universities, and cultural events were planned to realize the screening. Sociodemographic variables of gender, age, country of origin, and educational level, as well as sexual habits, were recorded for each patient. CT was detected via urine samples. An estimate of the prevalence of CT genital infection and its 95% confidence interval (CI) was made based on the exact binomial distribution, assuming that the sample is representative of the study population.

Results

A total of 628 subjects participated in the study, of whom 33 had a CT infection, giving a prevalence of 5.2% (95% CI: 3.6%, 7.3%). 93.9% of subjects with CT infection were female (p≤0.019) and 85% of the participants were Spanish nationals. Among vocational training students, the prevalence was 8.1%. Having had four or more sexual partners in the last month and in the previous year was significantly associated with CT infection (p<0.001).

Conclusion

Screening for CT genital infection in young sexually active women should be implemented in our country, as recommended by the various guidelines.

## Introduction

*Chlamydia trachomatis *(CT) genital infection has become the leading sexually transmitted infection (STI) in Europe in recent years [1]. Sexually active individuals aged 15-24 years remain the main risk and transmission group for genital CT infection [2]. In fact, women aged 20-24 years are one of the groups where it has increased the most [3]. One characteristic of this infection is that it can be asymptomatic in 70% of women and 50% of men. Women experience the most complications, as CT genital infection can lead to pelvic inflammatory disease and tubal infertility [4]. Currently, screening for CT in the asymptomatic population in Spain is carried out in individuals who are victims of sexual assault [5], women under 25 years of age who are pregnant [6], and other at-risk groups like sex workers or people diagnosed with HIV [7].

In Catalonia, between 2016 and 2019, the average annual increase in the number of cases was 79.2% [8]. However, the SARS-CoV-2 pandemic appears to have changed that trend. According to data from the Center for the Study of STIs in Catalonia (CEEISCAT), a 38.4% decrease in cases was reported in 2020 (99.7 cases per 100,000 inhabitants) [9]. COVID-associated confinements may have had an effect, and a recent study attributes the decline in the reporting of cases of various STIs to multiple causes [10]. Nevertheless, the rate of cases per 100,000 inhabitants increased again in 2021 [9].

Mathematical modeling suggests that implementing screening programs could reduce the prevalence of CT genital infection [11]. Screening of asymptomatic infected individuals has always been a subject of great controversy as to whether it is really cost-effective. In the article by Unemo et al. [12], it was questioned whether screening programs for this highly prevalent infection, especially among young people, were truly useful. In the latest European Centre for Disease Prevention and Control (ECDC) recommendations, it was found that screening in women could actually reduce the incidence of pelvic inflammatory disease by 36.5% each year (RR 0.6 [95% CI: 0.4-0.9]) [13]. However, the same report revealed that there was little evidence of the effectiveness and cost-effectiveness of screening programs in asymptomatic patients, although at that time only England and Germany had initiated screening programs [13].

Most studies recommend screening in certain situations [14], such as screening sexually active young individuals, which requires considerable monitoring resources and has not yet been implemented due to weak empirical evidence of effectiveness [15].

In Asturias in 2010, a study conducted in a young population found a prevalence of CT genital infection of 4.1% [16]. It was already considered that a screening program among young people could be useful for reducing the cases of CT and its complications in that population. Hence, we propose this study with the aim of determining the prevalence of CT among a young asymptomatic population and to evaluate whether a community screening program would be effective in reducing the number of cases.

## Materials and methods

Study design and participants 

This is a descriptive cross-sectional study examining the prevalence of consecutive inclusion of asymptomatic citizens aged between 18 and 25 years in Lleida, Spain, conducted between September 27, 2021, and May 7, 2022.

In September 2021 in Lleida, there were a total of 12,252 people aged between 18 and 25 years. According to the formula by Fleiss et al. [17], with a significance level of 0.05 and a beta risk of 0.2 in a bilateral contrast and a precision of 0.025, the target sample size was set at 890 people.

Inclusion criteria

All sexually active young individuals aged 18 to 25 years were eligible to participate. "Sexually active" was defined as individuals who had been sexually active within the previous six months.

Exclusion criteria

Unwillingness to sign the informed consent form or presenting with symptoms compatible with CT genital infection were grounds for exclusion.

Sample collection and procedures

We prioritized a sample comprising young people from various educational backgrounds, as we hypothesized that a sample with a high proportion of university students would not be representative and the results could underestimate the real prevalence of CT. We did not focus as much on gender because asymptomatic CT genital infections occur in both women and men.

To achieve the most representative sample of the young population aged between 18 and 25 years in the city, and to minimize selection biases, several campaigns were planned. Two campaigns were conducted during local music festivals (from September 27 to 30, 2021, and on May 6, 2022) to recruit participants from all educational backgrounds; two campaigns were held at university centers in the city (November 28, 2022, and March 20, 2022) to include university students; and additional campaigns took place in non-university training centers (from March 15 to 27, 2022) to include young people not attending university. Lastly, campaigns were held in social training centers in March 2022 to include individuals without secondary education and those with lower levels of education. During each campaign, basic training on CT genital infection was provided, young people were invited to participate in the study, and after completing a questionnaire on the digital platform, a urine sample was collected.

Post-campaign, the research team reviewed the results and contacted those young individuals with positive CT results to facilitate antibiotic treatment and perform contact tracing at the STI clinic of the regional hospital. Patients testing positive were treated between one week and 30 days later.

Variables and data collection

Sociodemographic variables recorded for each participant included gender, age, country of origin, and level of education. We established four gender categories: male, female, transgender, and other (including intergender, queer, bigender, etc.).

Data on sexual health and habits were gathered based on the CEEISCAT [18] epidemiological survey. Variables collected included sexual orientation, the number of sexual partners in the last month and year, condom use during sexual encounters, history of STIs in the last year, and the possibility of pregnancy. Participant data were collected through a survey using the European Commission's EuSurvey digital platform [19].

After the survey, a urine sample was collected to detect the presence of the following STIs: *Neisseria gonorrhoeae *(NG); *Trichomonas vaginalis *(TV); and *Mycoplasma genitalium* (MG). Additionally, due to the characteristics of the test, we evaluated the presence of typical commensal flora such as *Mycoplasma hominis *(MH), *Ureaplasma urealyticum* (UU), and *Ureaplasma parvum* (UP), microorganisms that have also increased in our region and may cause co-infections with CT. Participants testing positive for UU, UP, and MH received no treatment.

The method used for diagnosing CT genital infection and other STIs was the Allplex™ Seegene© Nucleic Acid Amplification Test (NAAT) STI Essential Assay. This test was performed on urine samples from both men and women and has a sensitivity of 88-95% and a specificity of 95-98% [20] for detecting CT infection. This test is approved and recommended by the Catalan Health Department.

Urine samples from cis-men and vaginal swabs from cis-women (either clinician- or self-collected) are considered the optimal specimen types for screening [21]. However, due to the nature of this study, which utilized asymptomatic community recruitment for reasons of practicality and acceptability, urine samples were collected for diagnosis. These samples were sent to the laboratory and stored in a refrigerator at temperatures between 4 and 8ºC until analyzed within 48 hours. Sample preparation was carried out as indicated by the manufacturer (Seegene), and DNA was extracted using EZ1 or QIASymphony (QIAGEN©) equipment. PCR detection of CT and other microorganisms was executed using the Allplex™ STI-7 V1-1 kit (Seegene©).

The amplification cycle threshold was determined using CFX96 software, following values recommended by the Seegene Allplex testing kit. A valid result required the sample to exhibit exponential growth with a sigmoid-shaped curve, enabling it to cross the cycle threshold.

Statistical analysis

An estimate of the prevalence of CT infection and its 95% confidence interval (CI), based on the exact binomial distribution, was performed. This estimate assumes that the sample is representative of the study population. Bivariate analyses were conducted based on the presence or absence of CT infection using the non-parametric Mann-Whitney test for continuous variables and Pearson's chi-square test for categorical variables. In instances where expected frequencies were lower than 5, Fisher's exact test was applied. Logistic regression analyses estimated crude odds ratios (ORs). For estimating adjusted ORs in a multivariable logistic regression model, the Boruta algorithm was previously applied to eliminate unimportant variables in predicting CT infection. Possible interactions with age and sex were evaluated, along with the calibration and discrimination of the final model using the Hosmer-Lemeshow test and the area under the ROC curve. Statistical analyses were carried out using R [22], applying a significance level of 0.05.

Ethical aspects

The study received approval from our institutional Research Ethics Committee (CEIC number 2520). Data were collected exclusively for research purposes and are maintained in compliance with Spanish Organic Law 3/2018 on Personal Data Protection and Regulation 2016/679 of the European Parliament.

## Results

A total of 628 people participated in the study, which represents 70.5% of the target sample. Of these, 33 had CT genital infection, a prevalence of 5.2% (95% CI: 3.6%, 7.3%). A description of the sample is given in Table 1. 

**Table 1:** Sociodemographic characteristics of the sample

	All Sample	No CT Infection	CT Infection	p-Value
	N=628	N=595	N=33	
Age (mean [95% CI])	20.0 [19.0;22.0]	20.0 [19.0;22.0]	20.0 [19.0;23.0]	0.508
Gender				
Man	166 (26.4%)	164 (98.8%)	2 (1.2%)	0.019
Women	445 (70.9%)	414 (93.0%)	31 (6.9%)	
Transgender	2 (0.31%)	2 (100%)	0 (0.0%)	
Other	15 (2.39%)	15 (100%)	0 (0.0%)	
Region of birth				
Spain	562 (89.5%)	531 (94.5%)	31 (5.5%)	0.9
South America	28 (4.46%)	27 (96.4%)	1 (3.5%)	
Other	38 (6.05%)	37 (97.4%)	1 (2.6%)	
Educational background				
Primary	20 (3.18%)	20 (100%)	0 (0.0%)	0.235
Secondary	20 (3.18%)	19 (95.0%)	1 (5.0%)	
Professional training	173 (27.5%)	159 (91.9%)	14 (8.1%)	
University or higher	415 (66.1%)	397 (95.7%)	18 (4.3%)	
Sexual orientation				
Heterosexual	427 (68.0%)	403 (94.4%)	24 (5.6%)	0.821
Bisexual	146 (23.2%)	139 (95.2%)	7 (4.7%)	
Homosexual	40 (6.37%)	39 (97.5%)	1 (2.5%)	
Unknown	15 (2.39%)	14 (93.3%)	1 (6.6%)	
Place of intervention				
Cultural events	175 (27.9%)	165 (94.3%)	10 (5.7%)	0.015
Universities	139 (22.1%)	138 (99.3%)	1 (0.7%)	
Professional study centers	153 (24.4%)	139 (90.8%)	14 (9.1%)	
Other educational centers	161 (25.6%)	153 (95.0%)	8 (4.9%)	

In all, 70.9% of the sample was female, 93.9% of the patients with CT infection were female (p≤0.019), and 85% of the participants were Spanish nationals. The majority of the sample were university students (66.1%). This is why most of the infected participants are university students (54% of those with CT infection). However, the prevalence of infection among university students was 4.3%, while among vocational students, the prevalence was 8.1%.

The distribution of participants was approximately 25% in each of the four campaigns (cultural activities, universities, professional training centers, and youth education centers). The highest number of subjects testing positive was found in professional training schools, with a prevalence of 9.1% (p=0.015), followed by cultural events.

Regarding sexual habits, a summary of the results can be found in Table 2. The number of sexual partners in the previous year (four or more) has a significant relationship with CT infection (p<0.001). Although 63.3% of the sample reported using barrier methods during sexual intercourse, 48% of the sample did not use any method in their last sexual encounter.

**Table 2:** Sexual habits of the sample The number of sexual partners in the previous year (four or more) has a significant relationship with CT infection (p<0.001).

	All Sample	No CT Infection	CT Infection	Overall p-Value	N
Sexual partners					
No permanent partner	339 (54.0%)	316 (93.2%)	23 (6.78%)	0.093	628
Permanent partner	289 (46.0%)	279 (96.5%)	10 (3.46%)		
Num. Sexual partners in last month (mean [95% CI])	1 [1.0;1.0]	1 [1.0;1.0]	1 [1.0;2.0]	<0.001	628
Num Sexual Partners in last year (mean [95% CI])	1 [1.0;3.0]	1 [1.0;3.0]	4 [2.0;7.0]	<0.001	628
Barrier methods					
Non-use of barrier methods	232 (36.9%)	216 (93.1%)	16 (6.90%)	0.22	628
Use of barrier methods	396 (63.1%)	379 (95.7%)	17 (4.29%)		
Non-use of barrier methods in last sexual relation	307 (48.9%)	287 (93.5%)	20 (6.51%)	0.228	628
Use of barrier methods in last sexual relation	321 (51.1%)	308 (96.0%)	13 (4.05%)		
Sexual work					
Not engaged in sexual work	621 (98.9%)	588 (94.7%)	33 (5.31%)	1	628
Has been a sexual worker	7 (1.11%)	7 (100%)	0 (0.00%)		
Previous STI					
Non-previous STI	589 (93.8%)	557 (94.6%)	32 (5.43%)	0.714	628
Previous STI	39 (6.21%)	38 (97.4%)	1 (2.56%)		
Pregnancy					
No possibility of pregnancy	618 (98.4%)	586 (94.8%)	32 (5.18%)	0.419	628
Possibility of pregnancy	10 (1.59%)	9 (90.0%)	1 (10.0%)		

Table 3 shows the results of the isolated microorganism detected in our sample and their co-infection with CT. Of the 33 CT infections, just one was isolated. The other 32 CT infections had a co-infection with other microorganisms.

**Table 3:** Co-infection and colonization of other microorganisms in patients with Chlamydia *Chlamydia trachomatis* (CT) co-infection was significantly associated with *Ureaplasma parvum* (UP) and *Mycoplasma hominis* (MH).

	All Sample	No CT	CT Infected	p-Value
	N= 628	N= 595	N= 33	
Neisseria gonorrhoeae	3 (0.5%)	3 (100%)	0 (0.0%)	1
*Trichomonas vaginalis*	1 (0.1%)	1 (100%)	0 (0.0%)	1
Mycoplasma genitalium	16 (2.5%)	15 (93.8%)	1 (6.2%)	0.583
Ureaplasma urealyticum	211 (33.6%)	195 (92.4%)	16 (7.5%)	0.095
*Ureaplasma parvum*	292 (46.5%)	270 (92.5%)	22 (7.5%)	0.027
*Mycoplasma homini*s	90 (14.3%)	77 (85.6%)	13 (14.4%)	<0.001

Estimation of crude ORs indicates that there is a higher risk of CT infection among women (OR=5.6, 95% CI: 1.7-38.2) and due to the number of sexual partners in the previous month (OR=1.2, 95% CI: 1.1-1.4) or in the previous year (OR=1.05, 95% CI: 1.0-1.1).

**Figure 1:**
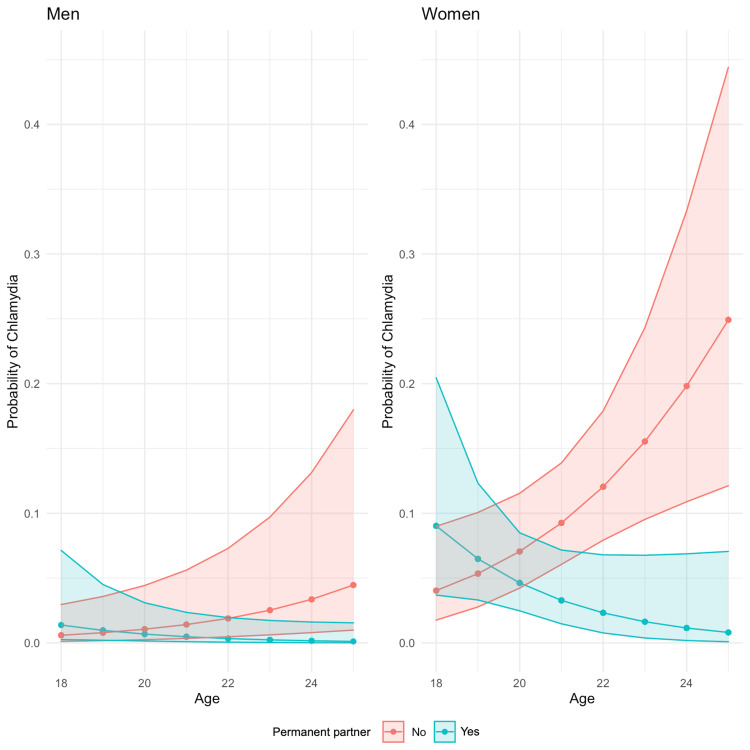
Multivariable logistic regression model, revealing an association with gender, age, and having or not having a steady partner Women are at higher risk of *Chlamydia trachomatis* (CT) genital infection than men. Furthermore, especially in women, age is associated with a higher risk of CT infection if they do not have a steady partner.

Figure 1 presents the results of the multivariable logistic regression model, revealing associations with gender, age, and the presence or absence of a steady partner. Women are at higher risk of CT genital infection than men. Moreover, in both men and women, but especially in women, age is associated with a higher risk of CT infection if they do not have a steady partner. Conversely, for those who do have a steady partner, age is associated with a lower risk of CT infection.

The Hosmer-Lemeshow test shows the acceptable calibration of the model, detecting no evidence of significant undercalibration. The discriminatory power measured by the area under the curve is 0.73 (95%CI: 0.6-0.8).

## Discussion

The prevalence of CT genital infection in our sample, composed of sexually active, young, asymptomatic people, is 5.2%. The infection mainly affects women, and an association has been detected with having had four or more sexual partners prior to sampling.

This figure is slightly higher than that in the 2010 study (4.1%) [16] but lower than in the preliminary study conducted by our team in 2018 [23], which was 7.4%. One might think that the pandemic, and its subsequent lockdowns over the past two years, may have reduced the reporting of cases, and therefore these figures would not reveal the real situation [10]. However, our team conducted an investigation [24] comparing data for urethritis during the first four months of the pandemic (March-June 2020), in which a significant reduction in the number of cases seen in our emergency departments was not detected. It will be important to continue to observe whether or not this trend persists.

What is clear, however, is that asymptomatic CT genital infection is much more common in women. In this screening program, 93.9% of those infected were women, although it is true that the majority of participants screened probably took part because multiple information campaigns have alerted young women to their sexual health.

The CDC has recommended screening for CT in sexually active young women since the early 1990s and has reiterated its recommendation in all guidelines released since then. The last recommendation was in 2021 [25]. In fact, our study suggests that it should be implemented because routine screening can detect many hidden infections, lead to their treatment, and avoid the resulting complications. Our screening project is novel because its design is community-centered, taking into account the social habits and different educational backgrounds of our target group: sexually active young people aged 18-25 years.

Another point of interest is that the most prevalent group of participants is vocational training students. We believe it is important to look for new communication strategies, as a recent review [26] showed that current messages do not translate into a better response, and campaigns with clear and real messages are needed.

In terms of sexual habits, 63% of the sample reported using barrier methods in their sexual relations, but only 51.1% have reported using a protective method in their last encounters. Condomless sexual relations have increased among some young populations [27]. This is likely because the fear of pregnancy has decreased with access to emergency contraception, and there is a perception that most STIs are harmless and HIV is a chronic disease [28].

This should lead us to profound reflection on how to address sexual health in the young population, as it is also the youngest patients who are most at risk, particularly women, due to the number of sexual partners and because they are in less stable relationships. It is also interesting to see that the older the patient is, the greater the risk if they do not have a permanent partner, in a population that should already be more knowledgeable about sexual health and more aware of the risks involved in unsafe sexual relations. In our study, we detected other microorganisms that may, in some circumstances, facilitate other STIs, some of them without clinical repercussions, but which confirm the low use of condoms. UP was detected in 46%, and UU in 33% of the samples. In fact, UP and MH infections were significantly associated with CT genital infection. This has also been described in other similar studies [29]. UP and MH infections have also been associated with complications such as infertility and should be treated appropriately if patients have symptoms [30]. In our study, we did not treat asymptomatic UP and MH patients.

Our study has several limitations, such as the fact that we did not achieve the total sample to attain the statistical power we desired, because we had difficulties recruiting people in some interventions. The majority of participants were female, which may affect the generalizability of the results and the low representation of the young population. However, one of its strengths is that the sample covers different socio-educational profiles of young people. Moreover, it is the first screening program for asymptomatic patients in the community carried out in our region and the first in our country in the last 10 years.

## Conclusions

We believe that screening for CT genital infection in young sexually active people, particularly women, should be implemented in Spain. ECDC guidelines recommend it, and the evidence of CT infection in our study corroborates it. Performing a free, annual urine determination of CT in all sexually active women under 25, could be a good strategy. With the collaboration of health centers and other entities, community-based screening, just like screening for HIV, could be implemented. We believe it is important to focus screening on certain risk groups, especially young women, to avoid the complications that a massive screening program may entail.

CT genital infection continues to increase and remains the most prevalent infection in Catalonia in young people, and we believe that establishing systematic screening in asymptomatic people and improving communication are two tasks that need to be addressed as soon as possible to avoid the complications of the spread of this infection, especially in women.
